# Pepper Fruit Elongation Is Controlled by *Capsicum annuum Ovate Family Protein 20*

**DOI:** 10.3389/fpls.2021.815589

**Published:** 2022-01-04

**Authors:** Yelena Borovsky, Amit Raz, Adi Doron-Faigenboim, Hanita Zemach, Eldad Karavani, Ilan Paran

**Affiliations:** Institute of Plant Science, Agricultural Research Organization, The Volcani Center, Rishon LeZion, Israel

**Keywords:** fruit shape, pepper (*Capsicum* spp.), ovate family protein, QTL mapping, natural variation in plants

## Abstract

Fruit shape is one of the most important quality traits of pepper (*Capsicum* spp.) and is used as a major attribute for the classification of fruit types. Wide natural variation in fruit shape exists among the major cultivated species *Capsicum annuum*, allowing the identification of several QTLs controlling the trait. However, to date, no genes underlying fruit shape QTLs have been conclusively identified, nor has their function been verified in pepper. We constructed a mapping population from a cross of round- and elongated-fruited *C. annuum* parents and identified a single major QTL on chromosome 10, termed *fs10*, explaining 68 and 70% of the phenotypic variation for fruit shape index and for distal fruit end angle, respectively. The QTL was mapped in several generations and was localized to a 5 Mbp region containing the ortholog of *SlOFP20* that suppresses fruit elongation in tomato. Virus-induced gene silencing of the pepper ortholog *CaOFP20* resulted in increased fruit elongation on two independent backgrounds. Furthermore, *CaOFP20* exhibited differential expression in *fs10* near-isogenic lines, as well as in an association panel of elongated- and round-fruited accessions. A 42-bp deletion in the upstream region of *CaOFP20* was most strongly associated with fruit shape variation within the locus. Histological observations in ovaries and fruit pericarps indicated that *fs10* exerts its effect on fruit elongation by controlling cell expansion and replication. Our results indicate that *CaOFP20* functions as a suppressor of fruit elongation in *C. annuum* and is the most likely candidate gene underlying *fs10*.

## Introduction

Fruit shape is one of the most important quality traits affecting consumer preference and breeder goals in vegetable crops. Typically, wild fruit is small and has a round shape, whereas intensive breeding following domestication has resulted in a wide diversity of sizes and shapes (van der Knaap and Ostergaard, 2017).

Tomato (*Solanum lycopersicum*) is the most important model plant species for studying fleshy fruit development and morphology (Seymour et al., 2013), and for which numerous quantitative trait loci (QTLs) controlling fruit shape variation have been identified (reviewed by van der Knaap et al., 2014; Wang et al., 2015a). *OVATE* was the first isolated gene underlying a tomato fruit shape QTL, and a null mutation in the gene resulted in pear-shaped fruit (Liu et al., 2002). *OVATE* was determined as a negative regulator of cell division and a suppressor of plant growth, because its overexpression resulted in smaller organs (Liu et al., 2002). Recently, another member of the *OVATE FAMILY PROTEIN*s (*OFP*), *SlOFP20*, was identified as underlying the tomato fruit shape QTL *suppressor of ovate1* (*sov1*; Wu et al., 2018). *OFP* homologs have been identified in diverse plant species (Liu et al., 2014; Snouffer et al., 2020) and have been associated with regulation of fruit shape in melon and tuber shape in potato (Wu et al., 2018). More recently, flat fruit of peach (*Prunus persica*) was also found to be associated with an inversion containing a member of the OFP family, *PpOFP1*, which is closely related to *Arabidopsis thaliana OFP1* and *SlOFP20*, where its expression was activated by the inversion (Zhou et al., 2020). Furthermore, *OFP* members have been associated with regulation of organ growth in rice (Xiao et al., 2017; Yang et al., 2018) and *Arabidopsis* (Li et al., 2011; Zhang et al., 2016), as well as with variation in plant architecture and response to abiotic stresses and hormones (Snouffer et al., 2020). Using a yeast two-hybrid screen, TONNEAU1-recruiting motif (TRM) proteins were identified as interacting with OVATE and SlOFP20 (Wu et al., 2018). TRM *LONGIFOLIA* homologs have been associated with variation in leaf and grain morphology in *Arabidopsis* and rice, respectively (Lee et al., 2006; Wang et al., 2015b). Collectively, these results indicate the involvement of the OFP–TRM module in controlling diverse aspects of plant organ morphology in multiple plant species.

A second gene family associated with control of fruit shape is IQ67 domain (IQD). The tomato fruit shape QTL *SUN* belongs to this family and mediates fruit elongation primarily by controlling cell-division patterns (Xiao et al., 2008; Wu et al., 2011). Interestingly, *sun* is a gain-of-function mutation mediated by inserting a retrotransposon near the locus. Association of IQD homologs and fruit shape has also been demonstrated in the cucurbits (Pan et al., 2017; Dou et al., 2018). In addition to *SUN*, *OVATE*, and *SlOFP20*, which were identified as underlying tomato fruit elongation QTLs, a fourth locus, *fs8.1*, has been fine-mapped to tomato chromosome 8 but the underlying gene has not yet been identified (Sun et al., 2015). The involvement of *FRUITFULL-like* MADS-box gene in regulating fruit elongation was demonstrated in cucumber (Zhao et al., 2019), but its function in regulating Solanaceae fruit shape is not known. *LOCULE NUMBER* (*LC*) and *FASCIATED* (*FAS*) synergistically control flat fruit shape and locule number, and have a pleiotropic effect on fruit weight and shape (Cong et al., 2008; Huang and van der Knaap, 2011; Munos et al., 2011). *WUSCHEL*, which is required for stem cell identity in meristems, underlies the *lc* mutation, whereas *CLAVATA3* underlies the *fas* mutation (Munos et al., 2011; Xu et al., 2015). Together, these genes control various developmental aspects, such as cell division, number of carpels, meristem size, and organization, which contribute to the morphological diversity of the fruit (van der Knaap and Ostergaard, 2017).

Pepper (*Capsicum* spp.), a member of the Solanaceae family, exhibits vast natural variation in fruit shape that is utilized for breeding of diverse fruit types. The genetic and molecular bases of the natural variation in pepper fruit shape have been analyzed mainly by QTL mapping studies (Ben Chaim et al., 2001, 2003a,b; Rao et al., 2003; Zygier et al., 2005; Barchi et al., 2009; Borovsky and Paran, 2011; Yarnes et al., 2013; Han et al., 2016; Chunthawodtiporn et al., 2018; Du et al., 2019; Lee et al., 2020). These studies detected two major fruit elongation QTLs, *fs3.1* and *fs10.1*, on chromosomes 3 and 10, respectively, and additional, more minor QTLs on multiple chromosomes. Analysis of genomic population structure in *C. annuum* revealed multiple fixed regions that are conserved in non-pungent varieties and partly overlap with known QTLs for fruit weight and shape in tomato (Hill et al., 2017). Furthermore, based on expression and QTL colocalization, candidate genes putatively associated with fruit morphology were identified (Hill et al., 2017). Genome-wide association study (GWAS) of 220 *C. annuum* accessions revealed four loci associated with fruit shape attributes, including a non-synonymous mutation in the gene *LONGIFOLIA1-like* on chromosome 3 (Colonna et al., 2019). Furthermore, a pepper homolog of *OVATE* was shown to be associated with fruit shape variation by downregulation using virus-induced gene silencing (VIGS; Tsaballa et al., 2011). However, it is not known whether this gene is associated with the natural variation in pepper fruit shape. Thus, to date, no genes underlying fruit weight and shape QTLs have been conclusively identified or their function verified in pepper. The objective of the present study was to dissect the phenotypic, genetic, and molecular bases for the extreme natural variation in *C. annuum* fruit shape. QTL mapping enabled the identification of a major fruit shape QTL, *fs10*. We provide evidence for the pepper ortholog of tomato *SlOFP20* underlying *fs10* and describe the cellular mechanism by which *fs10* exerts its effect on regulation of fruit shape.

## Materials and Methods

### Plant Material and QTL Mapping

For QTL mapping of fruit shape, we constructed an intraspecific *C. annuum* F_2_ population from the cross of two parents that exhibit extreme variation in fruit shape. Inbred line 1901 (long sweet CGN23289, kindly provided by Dr. A. Bovy, Wageningen University, Netherlands) has very long and narrow fruit and inbred line 5226 has small round fruit. For initial mapping, an F*_2_* population of 240 progenies was grown in the greenhouse and measured by digital caliper for fruit maximal length and width, fruit shape index (length/width), and the angle of the distal end at 5% from the fruit tip by Tomato Analyzer software version 3.0.^1^ For fine mapping of the QTL, selected F_2_ plants were self-pollinated until the F_4_ generation in which markers in the vicinity of the QTL were fixed for the parental alleles. For construction of QTL near-isogenic lines (QTL-NILs), selected F_4_ plants that were heterozygous at the most closely linked marker to the QTL (130 Mbp) were self-pollinated and fixed for alternate parental alleles in the F_5_ generation. All other chromosome 10 markers were monomorphic in the QTL-NILs.

We employed bulked segregant analysis using RNA sequencing (BSAseq) for QTL mapping by constructing bulks based on the phenotypic distribution of the F_2_ population (Borovsky et al., 2019). Three bulks composed of 13–15 plants per bulk for each of the phenotypic extremes were constructed and RNA was extracted from ovaries at anthesis for each of the six bulks. Total RNA was extracted using the GeneElute™ Mammalian Total RNA Extraction Miniprep Kit (Sigma). Genomic DNA was removed by DNaseI (Sigma) treatment. RNA sequencing was performed on a lane of 60-bp single-end reads in an Illumina HiSeq 2500 System at the Weizmann Institute of Science, Israel.

Putative QTL peaks were identified by plotting the number of homozygous single-nucleotide polymorphisms (SNPs) for each phenotypic extreme across the genome. Then, specific SNPs derived from the RNAseq data were genotyped in the entire F*_2_* population (Supplementary Table S1) and QTL mapping was performed using MapQTL software version 5^2^ with the interval mapping and Multiple QTL Model (MQM) functions. To study the interaction between *CaOFP20* and *CaOVATE*, we used an F_3_ population of 154 individuals and two-way ANOVA in JMP V.14.

To study the expression pattern of the QTL candidate gene in a wide pepper germplasm, we made use of the pepper G2P-SOL core collection that consists of 450 accessions representing the global diversity of this species.^3^ Based on preliminary visual assessment of the fruit shape index, we chose a subset of 30 *C. annuum* accessions representing elongated and round fruit. We further employed association mapping of fruit shape index and shape categories in a panel of 286 accessions from the G2P-SOL core collection. Fruit shape categories were determined by visual assessment of the fruits. For GWAS, we used the genotyping-by-sequencing (GBS) markers described in Tripodi et al. (2021), as well as specific DNA polymorphisms at the QTL candidate gene (Supplementary Table S1).

SNP calling for an association mapping population of 286 pepper accessions was carried out by Tassel 5.0 GBS v2 pipeline^4^ using the genome reference of Zunla-1 *C. annuum* L.^5^ and BWA software. The proportion of taxa with a genotype (minimum locus coverage) was set to 0.1 and minor allele frequency (MAF) was 0.01. The GWAS was performed using the mixed linear model by including kinship matrix in TASSEL 5.2.57 (Bradbury et al., 2007).

### Transcriptome and SNP Analyses

Raw reads from ovary RNA sequencing of the bulks were subjected to a filtering and cleaning procedure as described by Borovsky et al. (2019). Gene abundance was estimated using Cufflinks (Trapnell et al., 2010, v. 2.2) combined with gene annotations from the Pepper Genome Platform.^6^ Gene-expression values were computed as FPKM. Differential expression analysis was performed using the DESeq2 R package (Love et al., 2014). Genes with an adjusted *p*-value of no more than 0.05 were considered differentially expressed. The gene sequences were used as a query term for a search of the NCBI non-redundant (nr) protein database that was carried out with the DIAMOND program (Buchfink et al., 2015). Homologous sequences were also identified by searching against the Heinz 1706 *S. lycopersicum* genome v.3^7^ with the BLAST tool and an *E*-value threshold of 10^−5^. The search results were imported into Blast2GO version 4.0 for gene ontology (GO) assignments. The GO-enrichment analysis was carried out using the Blast2GO program based on Fisher’s Exact Test with multiple testing correction of false discovery rate (FDR). KOBAS 3.0 tool^8^ was used to detect the statistical enrichment of differentially expressed genes in the KEGG pathway and GO analysis.

For transcriptome SNP analysis, mapped bam files were preprocessed prior to performing SNP identification with Samtools/Picard Tools (Li et al., 2009)^9^ to mark duplicates, sort and add read groups. We performed local realignment to minimize false positives during the SNP-calling procedure (McKenna et al., 2010). Then, we performed genotype calling using Genome Analysis Toolkit, (GATK v.3.7) with the HaplotypeCaller option.

Expression levels of specific genes were determined by quantitative (q) RT-PCR in a Rotor-Gene 6000™ thermal cycler (Corbett Research, Australia) with at least five biological replications. Expression levels were normalized relative to *CaUbiquitin* (DQ975458.1). The PCR primers are listed in Supplementary Table S1.

### VIGS Experiments

A 296-bp fragment of *CA10g10680* amplified by primers OFP-Kpn-F and OFP-Xba-R (Supplementary Table S1) was cloned into the pTRV2 vector previously digested with the restriction enzymes *Kpn*I and *Xba*I. Empty vector pTRV2 served as a negative control. As a positive control, we used pTRV2:*CaPDS*, which contains the phytoene desaturase sequence and induces a photobleaching phenotype. The vectors were used to transform *Escherichia coli* DH5α competent cells, which were then plated on selective media (with 50 μg/ml kanamycin). After incubation at 37°C for 18 h, colonies were PCR-screened for the presence of the modified vectors using pTRV2-specific primers (PYL156-F and PYL156-R, Supplementary Table S1). Plasmid DNA from positive transformant colonies was isolated, digested with *Kpn*1 and *Xba*I, and analyzed on an agarose gel to check for fragment release from the pTRV2 vector. Plasmids from positive colonies were sequenced to verify successful vector construction.

For *Agrobacterium* infiltration, pTRV1, pTRV2, and the recombinant plasmids (pTRV2:*CaOFP20* and pTRV2:*CaPDS*) were transformed into *Agrobacterium tumefaciens* strain GV3101 using the freeze–thaw method. A 4-ml culture of each construct was grown at 28°C for 2 days in YEP medium containing 50 μg/ml kanamycin and 30 μg/ml gentamicin. The cultures were then diluted with YEP medium containing acetosyringone (0.01%, 200 mm), MES (1.0%, 1 M) and kanamycin and incubated at 28°C with shaking at 200 rpm overnight. On day of treatment, the cultures were centrifuged at 4000 rpm for 8 min, the supernatants were discarded and the pellets were resuspended in MMA medium [2.0% sucrose, 0.5% MS salts without vitamins, 1.0% MES (1 M), 0.1% acetosyringone (200 mm)] depending on the cell density to ensure OD_600_ = 0.8. Prior to the treatment, cell solutions were incubated in the dark for 4 h at room temperature.

Pepper seedlings were germinated in a speedling tray for 20 days before *Agrobacterium* infiltration. At the time of infiltration, the seedlings (20 each for negative and positive controls and 40 for gene silencing) had fully developed cotyledons while true leaves had only initiated. One day prior to infection, the seedlings were moved into a growing room with long-day conditions (16 light, 8 dark) at 16°C. The leaf infiltration media contained the pTRV1 plasmid and each of the pTRV2 plasmids mixed in a 1:1 ratio. For each seedling, both cotyledons were infiltrated with the prepared solutions using a 1-mL needleless syringe. Two weeks after inoculation, the seedlings were transplanted into 500-mL plastic pots and continued to grow under long-day conditions at 25°C, for a total of 7–8 weeks. Flowers were labeled at anthesis; 3 weeks after anthesis, fruit were removed and measured for fruit shape, and the tissues were harvested for RNA extraction to determine gene-expression level.

### Histology

Fresh samples of ovaries at anthesis and fruit pericarp at 3 weeks after anthesis were collected from the QTL-NILs and stored in FAA solution for 24 h. For histological analyses, samples were processed as described by Singh et al. (2019). Briefly, samples were dehydrated in an ethanol dilution series, embedded in paraffin wax, and sectioned with a microtome (Leica RM2245, Leica Biosystems, Germany). For ovaries and pericarps, 12 μm- and 20 μm-thick longitudinal and transverse sections, respectively, were made. The sections were then deparaffinized in Histo-Clear solution, rehydrated, and used for histochemical staining with Safranin Fast Green. Sections were examined under a light microscope (Leica DMLB) equipped with a Nikon DS-Fi1 digital camera. Image analyses for calculations of cell number, size, shape, and cross-sectional distances were done by the software ImageJ (NIH, United States) and NIS-Elements (Nikon).

Cell area, length, width, and shape index in the ovary and pericarp were measured in both the longitudinal and transverse sections. For longitudinal sections, the ovaries were divided into four zones, indicated by red lines in Supplementary Figures S1A,B. All cell parameters were measured in the four zones along the red lines in the ovary wall on one side of the ovary. Because the cell attributes were similar in zones 1, 2, and 3, the values for these three zones were averaged. For calculation of cell size in each zone, cells were counted in four squares of 100 μm × 100 μm in a grid in the middle of the ovary wall in areas without vascular tissue. Cell area was defined as the square area divided by the number of cells within the square. A total of 20 measurements were done for each zone in each NIL (five fruit per NIL, four squares per zone). Cell length, width, and shape index (length/width) were measured for 20 random cells per fruit in each zone in five fruit (a total of 100 cells per zone for each NIL). Cell attributes in the transverse sections were measured in zone 2 in the middle of the ovary. Cell number and distance across the ovary wall along the medio-lateral axis were measured along the red lines in the four zones. Cell number and distance along the proximal-distal axis of the entire longitudinal sections of the ovaries (black line in Supplementary Figures S1AB), as well as ovary maximum length, ovary maximum width, and ovary shape index were measured for intact ovaries. For the fruit pericarp, the same cell attributes were measured in three zones marked by red lines at 1/3, 2/3, and 3/3 positions (Supplementary Figure S1C). In each zone, a slice of 10 mm was excised from the pericarp and sectioning was done in the longitudinal direction on one side of the slice and in the transverse direction on the second side of the slice. For calculation of cell size in each zone, cells were counted in four squares of 500 μm × 500 μm in a grid in the middle of the pericarp in areas without vascular tissue.

## Results

### Phenotypic Characterization and QTL Mapping of *fs10*

**Figure 1:**
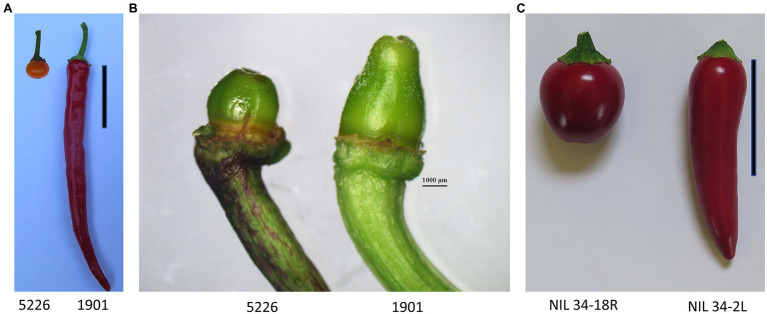
Fruit and ovaries of parents and near-isogenic lines (NILs) used in this study. **(A)** Ripe fruit of the mapping parents 5226 and 1901. **(B)** Ovaries at anthesis of the mapping parents 5226 and 1901. Ovary photos were taken by a Leica MZFLIII binocular. **(C)** Ripe fruit of the *fs10*-NILs 34-18R and 34-2 L. Scale bar = 5 cm.

To construct a QTL mapping population, we crossed line 1901 with an elongated fruit shape, and line 5226 with a small round fruit (Figure 1A; Table 1). Fruit shape index of the F_2_ progeny ranged from 0.9 to 6.4, with a mean of 2.5 indicating partial dominance of the round fruit shape. For initial QTL mapping, we employed BSA (Michelmore et al., 1991) using RNAseq of ovaries at anthesis from the two phenotypic extremes of fruit shape index in the F_2_ population. The mean fruit shape indices of the two bulks were 4.5 ± 0.9 and 1.3 ± 0.2 for the elongated- and round-fruited bulks, respectively. Anthesis was chosen for RNA extraction because it is a well-defined developmental stage at which differences in ovary shape are clearly observed (Figure 1B). In addition to fruit shape index which reflects the degree of fruit elongation, we measured the angle at the distal end of the fruit, which reflects the difference between elongated and round shape at that site (Table 1). The angles of the distal end of the F_2_ progeny ranged from 17.3^o^ to 160.2^o^ (Table 1). Values for fruit shape index and the angle of the distal end were strongly correlated (*r* = −0.83), suggesting common genetic control underlying these traits. Fruit shape index was highly correlated with fruit length (*r* = 0.93) and moderately correlated with fruit width (*r* = −0.5), indicating that fruit length is the predominant factor determining fruit shape index in the population.

**Table 1:** Fruit shape characteristics of parents, F_2_ progeny, and *fs10*-NILs.

	Genotype				
	1901	5226	Mean – F_2_	NIL 34-2 L	NIL 34-18R
Fruit length (cm)	18.3 ± 0.5	1.4 ± 0.04	4.7 ± 0.1	7.9 ± 0.5	1.9 ± 0.1^***^
Fruit width (cm)	1.7 ± 0.04	1.6 ± 0.09	2 ± 0.02	1.8 ± 0.1	2.2 ± 0.04^**^
Fruit shape index	12.7 ± 3.2	0.9 ± 0.05	2.5 ± 1.2	4.4 ± 0.3	0.9 ± 0.04^***^
Angle of the distal fruit end (^o^)	10.2 ± 2.2	186.3 ± 9.4	69.6 ± 32	26.9 ± 7.6	159 ± 23.9^***^

*Means ± SD. NIL 340-2 L and NIL 34-18R have elongated and round fruit, respectively*.

*** *Significant differences determined by t-test between the QTL-NILs at p < 0.001, and*

** *Significant differences determined by t-test between the QTL-NILs at p < 0.01*.

**Figure 2:**
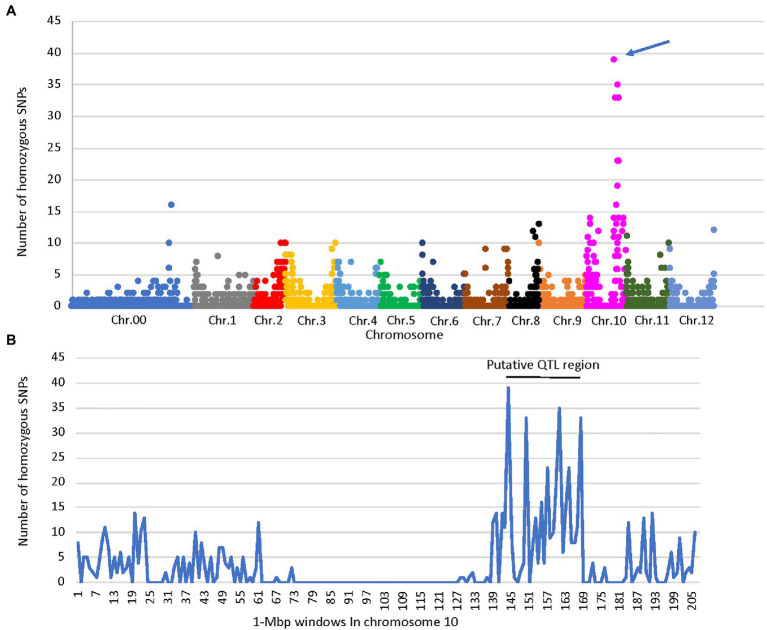
Distribution of homozygous SNPs identified by RNAseq of the elongated- and round-fruited bulks. **(A)** Distribution of SNPs on each chromosome in 1 Mbp windows, indicating a QTL peak marked by an arrow on chromosome 10. **(B)** Distribution of SNPs on chromosome 10 in 1 Mbp windows, indicating a putative QTL region at 142–169 Mbp. Chr.00 – SNPs that were not assigned to chromosomes.

**Figure 3:**
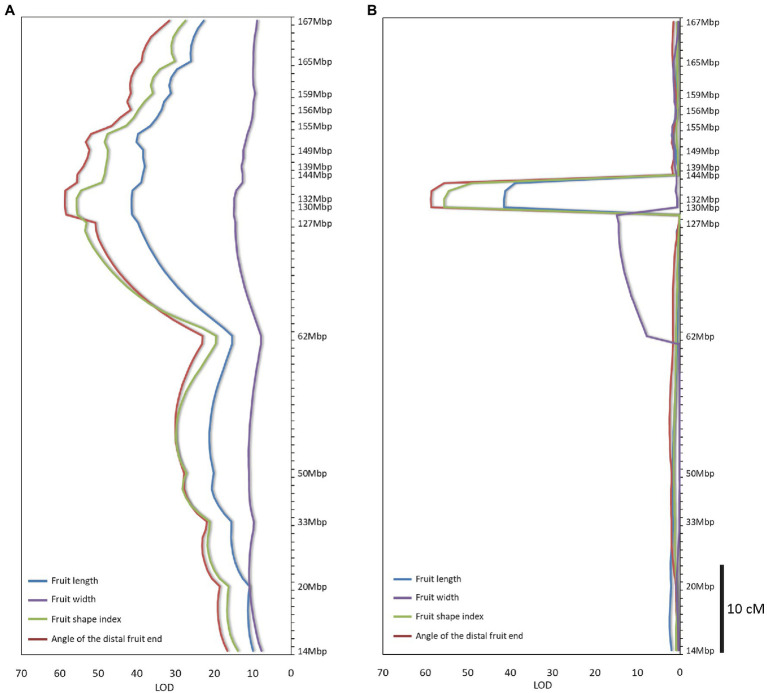
QTL mapping of fruit shape parameters in the F_2_ population. **(A)** Interval mapping. **(B)** MQM mapping. Significance threshold of LOD = 2.2 (*p* = 0.05) was determined for QTL detection by permutation test. Mapping analyses were performed by MapQTL software version 5 (Kyazma B.V., Netherlands).

A total of 2,517 homozygous SNPs were identified between the bulks (Supplementary Table S2). Plotting the number of homozygous SNPs differentiating the bulks across the genome indicated a major peak in a 27-Mbp region between 142 and 169 Mbp on chromosome 10 (Zunla genome, Qin et al., 2014; Figures 2A,B). Several additional minor peaks were identified at the beginning of chromosome 10 and across the genome which may represent minor QTLs affecting the trait (Figure 2A). We genotyped the F_2_ population with SNP markers derived from the RNAseq data flanking the putative QTL (Supplementary Table S1), and use of MapQTL software and MQM mapping enabled the identification of a single major QTL in the interval of 127–144 Mbp termed *fs10*, 130–132 Mbp being the most significant markers for fruit length, fruit shape index, and angle of the distal fruit end (Figures 3A,B; Table 2).

**Table 2:** MQM mapping of fruit shape attributes in F_2_ progeny of cross 5226 × 1901.

Trait	Marker at QTL peak	LOD	% Variation explained	Means^*^		
				AA	AB	BB
Fruit shape index	130–132 Mbp	55.6	67.9	1.3 ± 0.2	2.1 ± 0.5	3.9 ± 0.9
Fruit length (cm)	130–132 Mbp	41.4	56.9	2.5 ± 0.6	4.2 ± 1.1	7.3 ± 1.8
Fruit width (cm)	127 Mbp	14.8	26.1	2.2 ± 0.2	2.1 ± 0.3	1.7 ± 0.2
Angle of the distal fruit end (^o^)	130–132 Mbp	58.7	70	110.8 ± 21	67.1 ± 17.8	36.3 ± 10.9

* *AA – homozygous for 5226 allele, AB – heterozygous, and BB – homozygous for 1901 allele*.

To verify the mapping results based on the F_2_ generation and to further fine map the QTL, we fixed recombinant progeny in the F_4_ generation and phenotyped them for fruit shape index (Table 3). The data consistently indicated that the marker at 130 Mbp is the most tightly linked to the QTL because all of the lines possessing the 1901 allele at this marker had elongated fruit and all of the lines possessing the 5226 allele at this marker had round fruit. Line 16 allowed delineating the QTL to a 5-Mbp region between 127 and 132 Mbp.

**Table 3:** Marker genotypes and fruit shape index (FSI) of fixed recombinant lines and the mapping parents in the *fs10* region^*^.

Line	60 M	127 M	130 M	132 M	139 M	144 M	149 M	155 M	156 M	159 M	165 M	167 M	FSI
1901	3	3	3	3	3	3	3	3	3	3	3	3	12.7 ± 0.4 A
152	3	3	3	3	3	3	3	3	3	3	3	3	5.01 ± 0.1 B
16	1	1	3	1	3	3	3	3	3	3	3	3	3.7 ± 0.2 BC
135	3	3	3	3	3	3	1	1	1	1	1	1	3.0 ± 0.2 BC
78	3	3	3	3	1	1	1	1	1	1	1	1	2.8 ± 0.1 C
18	1	1	1	1	3	3	3	3	3	3	3	3	1.3 ± 0.2 D
38	3	3	1	1	1	1	1	1	1	1	1	1	1.0 ± 0.3 D
53	3	1	1	1	1	1	1	1	1	1	1	1	0.8 ± 0.3 D
126	1	3	1	1	1	1	1	1	1	1	1	1	0.8 ± 0.3 D
98	1	1	1	1	1	1	1	1	1	1	1	1	0.8 ± 0.2 D
5226	1	1	1	1	1	1	1	1	1	1	1	1	0.8 ± 0.4 D

* Genotypic scores of 1 and 3 represent homozygous alleles for the mapping parents 5226 and 1901, respectively. Markers used for mapping are coded by their physical map position in Mbp based on the Zunla genome (Qin et al., 2014). Differences among FSI means were determined by Tukey–Kramer range test at *P* < 0.05 and are indicated by different uppercase letters.

For further characterization of *fs10*’s effect, we constructed NILs in the F_5_ generation that were fixed for contrasting alleles at the 130 Mbp marker (Figure 1C; Table 1). Flower organs and leaf shape of the NILs were measured at anthesis, and exhibited significant differences in the shape indices of the ovary, anther, style, and petals (Supplementary Table S3). These indicated that *fs10* affects the shape of multiple flower organs and that organ shape differences are determined at early stages of flower differentiation prior to anthesis. No difference in leaf shape was observed between the NILs.

### *CaOFP20* Is a Candidate for Underlying *fs10*

To identify candidate genes underlying the QTL, the 5-Mbp region flanking *fs10* was examined for gene content (Zunla reference genome, Qin et al., 2014) as well as for differentially expressed genes based on transcriptome analysis of the F_2_ bulks. The QTL region contains 14 genes (Table 4), and the most likely candidate is *Capana10g001230*, the ortholog of *SlOFP20* that controls fruit elongation in tomato (Wu et al., 2018). Because only a partial sequence of *Capana10g001230* was available, we obtained the full-length sequence from the homologous gene *CA10g10680* of the reference genome CM334 V.1.55 (Kim et al., 2014). Two genes were differentially expressed (*Padj* ≤ 0.05, Table 4) between the F_2_ bulks: *Capana10g001225* coding for a non-specific lipid transfer protein (nsLTP) and *Capana10g001244* coding for a transmembrane protein with unknown function. Neither gene is known for its involvement in fruit shape control in plants. Interestingly, *Capana10g001230* was not differentially expressed between the bulks.

**Table 4:** Genes contained within the 5-Mbp *fs10* region and their expression in RNAseq of the F_2_ fruit shape index bulks.

Gene	Start	End	Fold change^*^	*Padj*	Zunla annotations
Capana10g001216	127792259	127817251		0.53	Unknown
Capana10g001219	128107003	128109679		no data	ATP synthase subunit a
Capana10g001220	128628965	128629689		no data	WRKY transcription factor
Capana10g001221	128733355	128734634		0.67	Unknown
Capana10g001225	129519501	129520422	1.02	1.22E-22	Non-specific lipid transfer
Capana10g001226	129689709	129690259		no data	Unknown
Capana10g001230	130159585	130160583		0.82	Ovate family protein^**^
Capana10g001234	130938359	130939294		no data	E3 ubiquitin-protein ligase
Capana10g001235	130939861	130946835		0.97	B3 domain-containing transcription repressor
Capana10g001238	131224816	131227923		no data	Translation elongation factor
Capana10g001241	131995454	131998780		0.54	Serine/threonine protein kinase
Capana10g001243	132048237	132055413		0.55	Vacuolar-sorting receptor
Capana10g001244	132155950	132166572	−1.02	0.0001	Transmembrane protein 87B
Capana10g001247	132428230	132429811		0.38	Signal recognition particle

* log2 fold change (fruit shape elongated bulk/fruit shape round bulk).

** CM334 v. 1.55 annotation.

### *CaOFP20* Is Differentially Expressed in the QTL-Nils

Because we knew that *SlOFP20* is involved in controlling tomato fruit shape, we further focused on the role of its ortholog *Capana10g001230* (termed *CaOFP20*) in determining fruit shape variation in pepper. Sequencing the open reading frame (ORF) of the gene in the parents of the mapping population revealed a single non-synonymous K131E amino acid substitution leading to a change of lysine (basic amino acid) in 1901 to glutamic acid (acidic amino acid) in 5226. This substitution is not included within the conserved OVATE domain at the C terminus of the protein (Liu et al., 2002).

**Figure 4:**
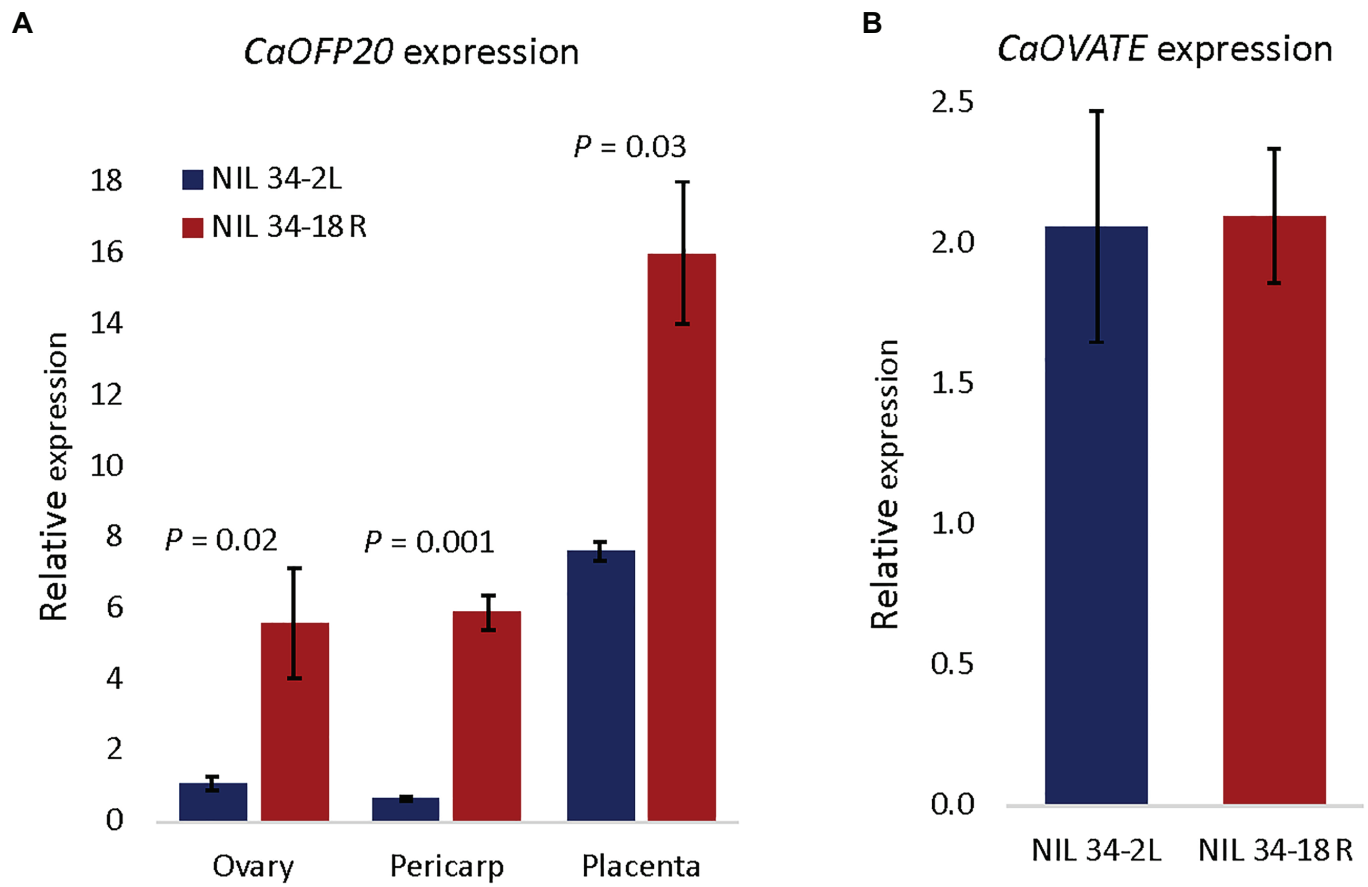
Expression of *CaOFP20* is downregulated in the elongated fruit *fs10*-NIL 34-2L, whereas no difference was observed for *CaOVATE*. **(A)** Expression of *CaOFP20* in ovary at anthesis and in pericarp and placenta of the fruit 3 weeks after anthesis. **(B)** Expression of *CaOVATE* in ovary at anthesis. Significant differences between the NILs were determined by Student’s *t*-test.

Examination of the expression pattern of *CaOFP20* throughout flower and fruit development in line 6421^10^ (Liu et al., 2017) indicated that the gene is expressed at its highest level during flower development at anthesis but is also highly expressed at later stages of fruit pericarp and placenta development (Supplementary Table S4). We therefore measured its expression in three tissues of the QTL-NILs: ovary at anthesis, and fruit pericarp and placenta at 3 weeks after anthesis. In all three tissues, *CaOFP20* exhibited significantly higher expression in the round-fruited NIL compared to the elongated-fruited one (Figure 4A), consistent with the role of *SlOFP20* as a suppressor of fruit elongation in tomato (Wu et al., 2018).

Because *SlOFP20* had been identified as underlying the *suppressor of ovate* (*sov1*) mutation in tomato, we determined whether the expression of the pepper *OVATE* ortholog (*CaOVATE*, *Capana02g002672*) is altered in the NILs; no difference in expression level of this gene was observed at anthesis (Figure 4B). Very low levels of expression of *CaOVATE* were observed in the pericarp and placenta. To further assess the relationship between *CaOFP20* and *CaOVATE* in determining fruit shape index, we scored both markers in the F_3_ generation and tested their possible interaction by two-way ANOVA. While *CaOFP20* had the expected major effect on fruit shape index (*p* < 0.0001), *CaOVATE* had a minor effect on the trait (*p* = 0.02), and the interaction was not significant (*p* = 0.06).

### Silencing of *CaOFP20* by VIGS Is Associated With Fruit Elongation

**Figure 5:**
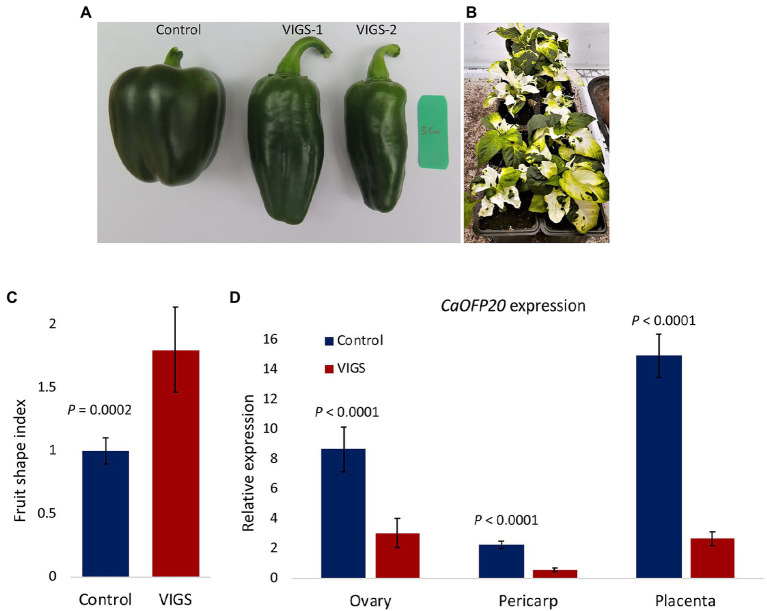
Silencing of *CaOFP20* in cv. Maor by VIGS. **(A,C)** Silencing of *CaOFP20* results in fruit elongation. Control – inoculation with empty vector. VIGS-1 and VIGS-2 are examples of silenced fruit 3 weeks after anthesis. **(B)** Silencing *CaPDS* as a positive control. Silenced plants did not set fruits. **(D)** Expression of *CaOFP20* is downregulated in silenced elongated fruit. Significant differences between the silenced and control fruit were determined by Student’s *t*-test.

To determine whether *CaOFP20* underlies fruit shape variation, we employed VIGS to knock down its expression in the blocky fruited cv. Maor and changes in fruit morphology were examined 3 weeks after anthesis. Out of 20 fruit examined, 7 exhibited an elongated shape (Figures 5A,B). Silenced elongated fruits were longer and narrower than the control fruit (inoculated with empty vector), resulting in a mean fruit shape index of 1.8 ± 0.3 and 1.0 ± 0.1 for the elongated and control fruit, respectively. Expression of *CaOFP20* was examined by qRT-PCR in ovaries at anthesis and in fruit pericarp and placenta tissue 3 weeks after anthesis. In all three tissues, *CaOFP20* exhibited significantly lower expression levels in the elongated genotypes (Figure 5C).

**Figure 6:**
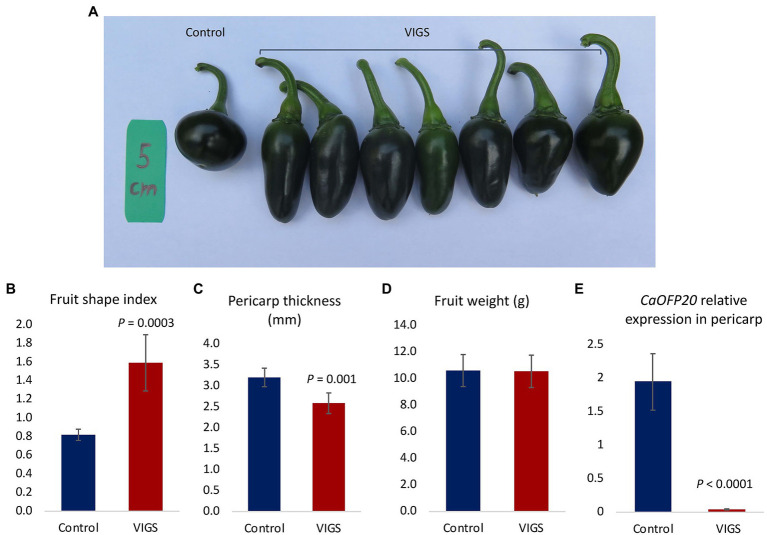
Silencing of *CaOFP20* in line 4590 by VIGS. **(A)** Silencing of *CaOFP20* results in fruit elongation. Control – inoculation with empty vector. VIGS – examples of silenced fruit 3 weeks after anthesis. **(B–D)** Fruit shape index, pericarp thickness, and fruit weight of silenced and control fruit. **(E)** Expression of *CaOFP20* is downregulated in silenced elongated fruit. Significant differences between the silenced and control fruit were determined by Student’s *t*-test.

To test the effect of silencing *CaOFP20* on the background of a round fruit, we employed VIGS on the round-fruited line 4590. Out of 20 fruit examined, 7 had an elongated shape (Figure 6A). Silenced elongated fruits were longer and narrower than the control fruits (inoculated with empty vector), resulting in a fruit shape index of 1.59 ± 0.3 in the silenced fruit compared to 0.8 ± 0.06 in the control fruit (Figure 6B). Pericarp thickness was significantly thinner in the elongated fruit (Figure 6C), but no difference in fruit weight was observed (Figure 6D). Expression of *CaOFP20* in the pericarp 3 weeks after anthesis was significantly reduced in the elongated fruit (Figure 6E), similar to the silenced “Maor” fruit. Taken together, silencing of *CaOFP20* by VIGS provided evidence for this gene’s function as a suppressor of fruit elongation in blocky and round pepper.

### *CaOFP20* Is Associated With Natural Variation of Fruit Shape in Pepper Germplasm

**Figure 7:**
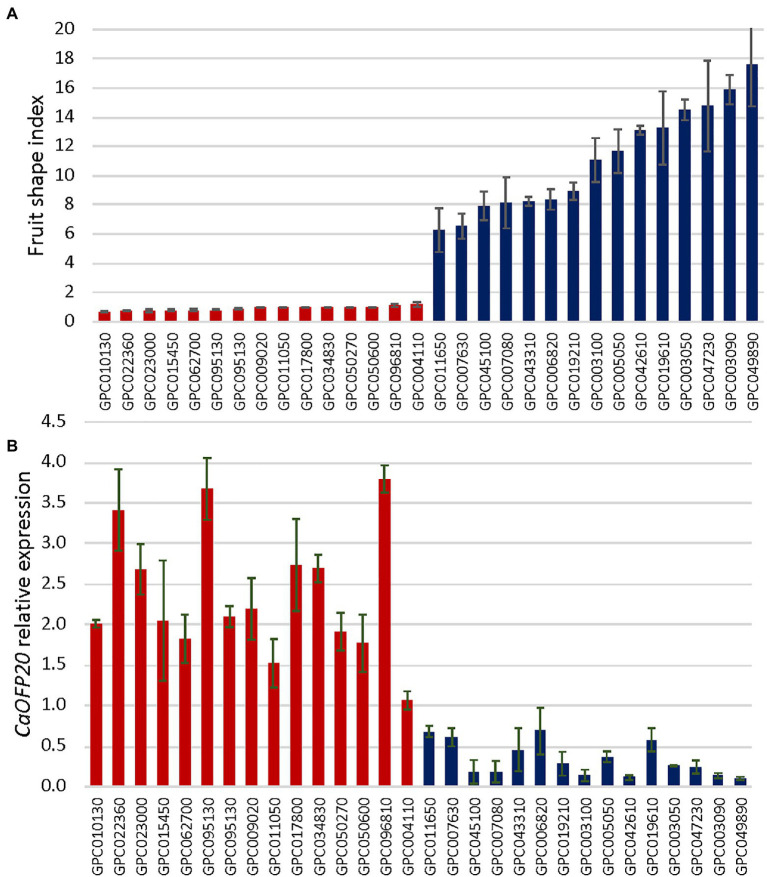
Fruit shape index is associated with expression level of *CaOFP20* in a panel of round- and elongated-fruited accessions. **(A)** Ripe fruit shape index of accessions in the panel. **(B)** Expression of *CaOFP20* in fruit pericarp 3 weeks after anthesis. Error bars represent standard deviation of three fruit per accession. Columns of round- and elongated-fruited accessions are colored in red and blue, respectively.

To test whether the expression pattern of *CaOFP20* is associated with natural variation in fruit shape, we selected 30 *C. annuum* accessions representing elongated and round fruit from the pepper G2P-SOL core collection. The mean fruit shape indices of the elongated- and round- fruited accessions were 11.09 ± 3.6 and 0.92 ± 0.13, respectively (Figure 7A). We performed qRT-PCR of *CaOFP20* using RNA from fruit pericarp tissues 3 weeks after anthesis. The expression level of the gene was significantly higher in the round-fruited accessions than in the elongated ones (Figure 7B), and expression level was strongly negatively correlated with fruit shape index (*r* = −0.82), indicating that *CaOFP20* is widely associated with fruit shape variation in *C. annuum*.

**Figure 8:**
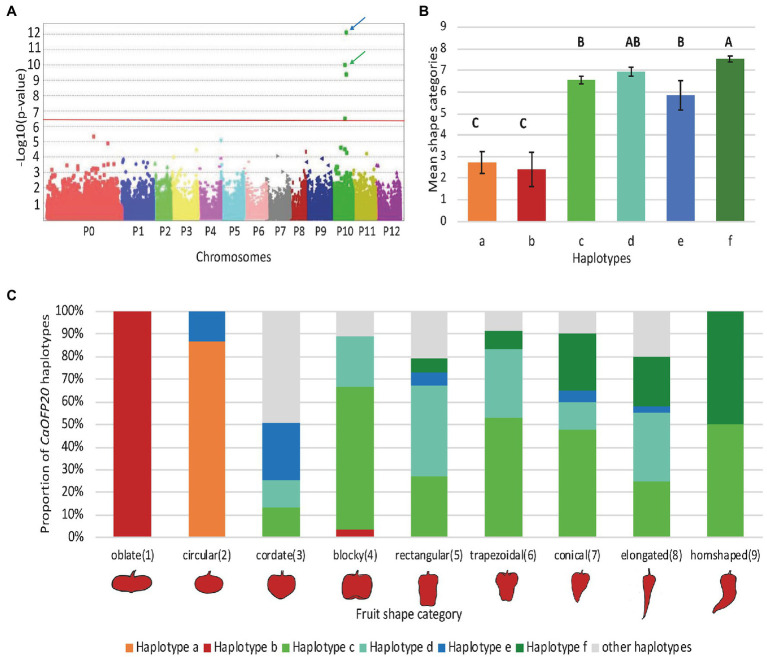
GWAS plot and haplotypes based on allelic variation at *CaOFP20* and their association with fruit shape categories in the association panel. **(A)** GWAS plot for fruit shape categories in the association panel. Blue arrow indicates the SNP at position 142898783 bp, downstream of *CaOFP20*; green arrow indicates the most significant marker at the *CaOFP20* locus at 130121672 bp. Red line represents the FDR-derived genome-wide significance threshold. P0 – SNPs that were not assigned to chromosomes. **(B)** Means of fruit shape categories of the different haplotypes. The six haplotypes in the association panel had at least five accessions per haplotype. Differences among means were determined by Tukey–Kramer range test at *p* < 0.05 and are indicated by different uppercase letters. **(C)** Haplotype composition of fruit shape categories.

To identify additional polymorphisms at the *CaOFP20* locus, we exploited the 30X resequencing genomes of the two parental lines of the mapping population as part of the G2P-SOL project. Within 50 kbp of the intergenic region upstream of *CaOFP20*, we detected several SNPs and a deletion of 42 bp at position 130121672 bp, 38381 bp upstream of the start codon of the gene in line 1901. Putative cis-acting elements could be found in the SNP regions as well as within the 42 bp InDel (Supplementary Tables S5, S6). Furthermore, sequencing the ORF of *CaOFP20* in the 30 elongated- and round-fruited accessions revealed five non-synonymous substitutions (Table 5). We developed molecular markers based on the promoter InDel and the ORF substitutions (Supplementary Table S1) and genotyped all 286 accessions. These genotypic data allowed us to identify six haplotypes consisting of at least five accessions per haplotype based on the *CaOFP20* locus (Table 6). Haplotypes a and b were clearly distinguished from the others in their effect on fruit shape, associated with circular and oblate shapes, respectively (Figures 8B,C). Haplotypes a and b had the same amino acid substitutions and differed in presence/absence of the promoter InDel (Table 6). Most of the accessions with circular fruit shape did not carry the 42-bp deletion, while all other accessions with various fruit shape categories, carried the 42-bp deletion. GWAS revealed a highly significant association of fruit shape categories with *CaOFP20* markers on chromosome 10 (Table 5; Figure 8A), whereas no association of *CaOFP20* markers was observed for fruit shape index (data not shown). Among the *CaOFP20* markers, the InDel marker was most strongly associated with fruit shape (*p* = 9.86 × 10^−11^, Table 5). Surprisingly, the most significantly associated marker with the trait in the panel was found at position 142898783 bp on chromosome 10 (*p* = 7.34 × 10^−13^), about 12 kb downstream of *CaOFP20*.

**Table 5:** InDel in the promoter of *CaOFP20* and non-synonymous substitutions in its coding region, and their significance level for fruit shape categories in the association panel.

	Upstream InDel	Non-synonymous substitutions				
		aa125	aa131	aa172	aa215	aa225
Position in genome^*^	130121672	130160428	130160443	130160567	130160695	130160728
Nucleotide InDel or substitution	GAGTTATATTAAAGTAAAGAAGG AGGGTTTTATGATACACCA	A/C	G/A	G/A	G/C	C/G
Amino acid substitution		Ser(S)/Arg(R)	Glu(Е)/Lys(K)	Lys(K)/Glu(Е)	Gln(Q)/Glu(Е)	Ser(S)/Arg(R)
GWAS association (P)	9.86 × 10^−11^	0.004	0.03	3.09 × 10^−5^	3.15 × 10^−7^	> 0.05

* Zunla genome (Qin et al., 2014).

**Table 6:** Haplotypes based on allelic variation at *CaOFP20* in the association panel.

Haplotype	Number of plants	42-bp deletion^*^	aa125	aa131	aa172	aa215	aa225
a	15	−	S	E	K	Q	S
b	12	+	S	E	K	Q	S
c	84	+	S	K	K	Q	S
d	58	+	R	E	E	E	R
e	13	+	S	E	E	E	R
f	43	+	S	E	E	E	S

* (+) and (−) indicate presence and absence of the 42-bp deletion, respectively.

### *fs10* Exerts Its Effect on Fruit Shape by Affecting Multiple Cellular Mechanisms

To decipher the cellular mechanisms underlying fruit shape variation conferred by *fs10*, we analyzed cellular parameters of *fs10*-NIL ovaries at anthesis and fruit pericarp tissue 3 weeks after anthesis. Longitudinal and transverse sections were performed in four and three zones in ovaries and pericarps, respectively (Supplementary Figures S1A–C). The total distance and total number of cells between the proximal and distal ends of the entire ovary in longitudinal section, marked along the black line (Supplementary Figures S1A,B), did not change significantly (Supplementary Table S7). In contrast, maximum ovary width and ovary shape index were greater in NIL 34-18R and NIL 34-2 L, respectively. Average cell area, cell length, cell width, cell shape index, number of cell layers between internal and external epidermis, and distance between internal and external epidermis in the ovary wall in longitudinal sections were significantly increased in NIL 34-18R. Similarly, these parameters, except for cell shape index, were significantly increased in transverse sections of the NIL 34-18R ovaries. These results indicate that at anthesis, ovary cells in the round-fruited NIL were larger and more numerous than in the elongated-fruited NIL. In pericarp longitudinal sections (Supplementary Figure S1C), the predominant factors were cell length and cell shape index, which were significantly increased in NIL 34-2 L in accordance with the difference in fruit elongation (Supplementary Table S8). In transverse sections, the thicker pericarp of NIL 34-18R had a larger cell area and greater number of cell layers between the internal and external epidermis tissues. Taken together, *fs10* exerts its effect on fruit shape by coordinately affecting multiple cellular mechanisms, including cell size, cell number, and cell shape.

### Transcriptome Analysis of RNA Bulks

Transcriptome analysis of the RNA bulks from ovaries at anthesis revealed 278 and 332 differentially upregulated and downregulated genes in the elongated-fruited vs. round-fruited bulk, respectively (Supplementary Table S9). GO analysis revealed significant enrichment of processes associated with photosynthesis, oxidation reduction, hormone metabolism, flavonoid synthesis, cell wall organization, protein binding, circadian rhythm, defense response, and fatty acid and lipid metabolism (Supplementary Figure S2). Hormone-enriched categories included response to auxin, auxin binding, auxin metabolism, cytokinin signaling, gibberellin (GA) signaling, response to GA, response to abscisic acid, response to jasmonic acid (JA), JA signaling and biosynthesis, and response to ethylene.

Thirty-five genes associated with cell wall organization were differentially expressed between the bulks, mostly upregulated in the round-fruited vs. elongated-fruited bulk and in accordance with the round-fruited NIL that had larger cells in the ovary (Supplementary Table S9). These genes coded for enzymes associated with biosynthesis, modification, and degradation of cell wall components and their involvement in cell wall extension, such as pectinesterase, beta-galactosidase, and polygalacturonase, and were either up- or downregulated in the round-fruited bulk. Two genes encoding extensin, a non-enzymatic protein associated with cell wall loosening, were upregulated in the round-fruited bulk. Additional genes associated with cell wall loosening were xyloglucan endotransglucosylase and expansin, which were downregulated in the round-fruited bulk.

GAs are plant hormones that control organ growth (Wang et al., 2017). Four genes associated with GA metabolism were differentially expressed between the bulks. Gibberellic acid 2-oxidase (GA2ox), associated with deactivation of GA, exhibited marked upregulation in the round-fruited bulk. Overexpression of GA2ox results in organ growth inhibition in several species, e.g., *Arabidopsis* and *Jatropha*, due to a decrease in endogenous bioactive GA (Hu et al., 2017). Similarly, 2-oxoglutarate and Fe(II)-dependent oxygenase, associated with structural modification of GA, were upregulated in the round-fruited bulk. In contrast, gibberellin 3-beta-hydroxylase 3, associated with GA activation, was upregulated in the elongated vs. round-fruited bulk.

Auxin is involved in the regulation of cell wall loosening and cell elongation (Majda and Robert, 2018; Wang et al., 2019). Eight genes associated with auxin signaling were differentially expressed between the bulks, mostly upregulated in the round-fruited bulk. These included several auxin response factors (ARFs), such as *ARF2*, *ARF6*, and *ARF8*. Mutations in *ARF2* and *ARF8* in *Arabidopsis* are associated with an elongated hypocotyl (Tian et al., 2004; Okushima et al., 2005). Other differentially expressed auxin-signaling genes were *TIR1* and *LAX1*, which function in auxin reception and transport, respectively. Additional genes associated with auxin biosynthesis (indole-3-acetamide hydrolase) and conjugation (indole-3-acetic acid-amido synthetase GH3.5) were upregulated in the elongated-fruited bulk.

Cytokinin is involved in the control of cell division and organ growth, among other developmental processes (Wybouw and De Rybel, 2019). Cytokinin hydroxylase catalyzes the biosynthesis of trans-zeatin and was upregulated in the round-fruited bulk vs. elongated-fruited bulk in this study. Trans-zeatin functions in shoot growth in *Arabidopsis* (Kiba et al., 2013), and in early tomato fruit development (Matsuo et al., 2012); however, its precise mode of action is not known.

JA is involved in the control of plant defense as well as plant growth under stress conditions. JA further interacts with other hormones involved in controlling plant growth, such as cytokinin, GA, and auxin (Jang et al., 2020). Six upregulated genes involved in JA biosynthesis and signaling were differentially expressed in the round-fruited vs. elongated-fruited bulk.

Ethylene acts as a regulator of plant growth, promoting cell expansion, and division (Dubois et al., 2018). Ethylene’s effect on cell expansion is mediated by the cell wall loosening expansins. Eleven genes in the round-fruited bulk, involved in ethylene biosynthesis and signaling, were differentially expressed, mostly upregulated.

## Discussion

### Mapping of *fs10*

To decipher the control of natural variation in fruit shape in *C. annuum*, we crossed two parents that exhibit diverse fruit types and extreme variation in fruit elongation. By combining BSAseq and map-based approaches, we detected a single major QTL, *fs10*, on chromosome 10 explaining 69% of the phenotypic variation for fruit shape index in the population. Additional minor QTLs may be present throughout the genome, as implied from the genome-wide homozygous SNP distribution; however, MapQTL analysis revealed only one significant QTL on chromosome 10. *fs10* likely corresponds to *fs10.1* which has been mapped in interspecific crosses of *C. annuum*×*C. chinense* (Ben Chaim et al., 2003a; Borovsky and Paran, 2011). The most closely linked marker to *fs10.1* was CT11 (Borovsky and Paran, 2011), corresponding to Capana10g001248 at position 132521846 in the Zunla genome, which is tightly linked to *CaOFP20*. Sequencing the ORF of *CaOFP20* in the EMS elongated-fruited mutant *E-1654* (Borovsky and Paran, 2011) did not reveal any mutation in the coding region of the gene. Thus, it is likely that another as yet unknown fruit shape gene is tightly linked to *CaOFP20*.

Interestingly, *CaOFP20* at 130 Mbp mapped outside the major peak identified by BSAseq in the 142–169 Mbp region of chromosome 10 (Figure 2B). This discrepancy likely results from a bias of SNP frequency along chromosome 10; the *CaOFP20* region had a very low content of polymorphisms compared to the high content of polymorphisms in the major peak area detected by BSAseq. Fine mapping using fixed recombinants clearly excluded the 142–169 Mbp region from containing the QTL (Table 3). It may be, however, that the peak discovered by the GWAS in the 142 Mbp region (Figure 8A) corresponds to an unknown QTL. Mining the 142 Mbp region for putative candidates did not reveal genes with known association to organ growth.

The parents of the mapping population and NILs differed not only in the degree of fruit elongation but also in the shape of the distal fruit tip; elongated fruit had more pointed fruit tips than the short and round fruit. QTL mapping and the high correlation between these two traits indicate that they are likely controlled by the same locus, suggesting that fruit elongation is associated with reduced angle of the distal end of the fruit. Compared to the parents of the mapping population that differed 13-fold in their fruit shape index, the QTL-NILs differed only 4-fold, and the maximal fruit shape index in the F_2_ generation differed only 2-fold. This indicates that additional unknown QTLs with low effects, as well as epistasis, likely contribute to the genetic control of the trait, as was demonstrated by the interaction of *SUN*, *ovate*, and *fs8.1* (Wu et al., 2015), as well as of *ovate* and *sov*, in tomato (Wu et al., 2018).

### *CaOFP20* Underlies *fs10* and Functions as a Repressor of Organ Growth

Fine mapping localized *fs10* to a 5-Mbp region that contains 14 genes, including *CaOFP20*. Due to prior identification of the tomato and potato *CaOFP20* orthologs underlying tomato fruit shape and potato tuber shape (Wu et al., 2018), as well as its differential expression between the QTL-NILs, we chose this gene as a primary target for gene silencing to test its function in pepper. VIGS knockdown of *CaOFP20* expression in both blocky and round-fruited backgrounds was associated with fruit elongation (Figures 5, 6). Unexpectedly, *CaOFP20* did not exhibit differential expression in the ovary transcriptome analysis of the bulks, likely due to masking of its expression by other unknown genes in the genome. However, qRT-PCR of the QTL-NILs using three tissues (ovary, pericarp, and placenta), as well as pericarp tissue from 30 diverse accessions, showed its higher expression level in the round-fruited accessions (Figures 4, 7).

Resequencing the genomes of the two parental lines revealed a 42 bp InDel about 38 Mbp upstream of *CaOFP20*. The 42-bp deletion was very strongly associated with fruit shape variation in the GWAS and is the most likely factor affecting reduction in the gene’s expression in elongated accessions, as no other structural variations were observed in the promoter region. Among the putative cis-acting regulatory elements found within the 42 bp InDel, the NTBBF1ARROLB motif (Supplementary Table S6) associated with auxin regulated expression may be affecting the expression of *CaOFP20*. Furthermore, sequencing the ORF of *CaOFP20* in 30 round- and elongated-fruited accessions revealed five SNPs causing non-synonymous amino acid substitutions. None of these substitutions was included in the conserved OVATE domain in the protein and it is not known whether they affect the protein’s function. Nevertheless, two of these substitutions, at aa 172 and aa 215, were strongly associated with fruit shape variation in the association panel (Table 5). Taken together, these results indicate that *CaOFP20* functions as a suppressor of fruit elongation in diverse *C. annuum* backgrounds, and it likely underlies *fs10*. Shape measurements of additional flower organs in the QTL-NILs indicated that the QTL suppresses growth of additional flower organs but has no effect on leaf growth. GWAS of the G2P-SOL panel revealed a significant association of *CaOFP20* markers with fruit shape categories, but not with fruit shape index. This indicates that the main effect of *CaOFP20* on fruit shape variation is the differentiation between elongated and round shapes, but not the magnitude of the fruit elongation.

Among the 14 genes located within the *fs10* QTL interval, two genes exhibited differential expression between the bulks. *Capana10g001225* was upregulated in the elongated-fruited bulk and codes for nsLTP, which belongs to a multigene family in plants that is involved in many physiological processes, such as response to biotic and abiotic stress, cutin and wax metabolism, and seed and pollen development (Liu et al., 2015). The potential involvement of nsLTP in cell wall organization and cell shape determination was shown in tobacco and *Arabidopsis* (Nieuwland et al., 2005; Ambrose et al., 2013). In tobacco, a nsLTP was identified that functions as a cell wall loosening protein in the pistil, likely associated with pollen tube growth. The tobacco gene (NCBI accession no. BAA03044) is highly homologous to *Capana10g001225*, as well as to an additional nine genes from the nsLTP family, seven of which are located in different regions of chromosome 10. Therefore, it is not possible to determine whether *Capana10g001225* and *BAA03044* are orthologous. Moreover, due to the high redundancy of the nsLTP family, it is not possible to downregulate *Capana10g001225* in a gene-specific manner by VIGS. Therefore, we cannot exclude this gene’s possible involvement in fruit shape regulation by cell shape determination.

### The Mechanisms of Fruit Shape Regulation by *fs10* in Pepper

Variation in fruit shapes of the QTL-NILs is already manifested at anthesis. At this stage, the ovary wall of NIL 34-18R is thicker due to increased cell size and number. The decrease in ovary shape index in NIL 34-18R results from expansion of the ovary wall along the medio-lateral axis. This effect is most pronounced at the distal end of the ovary, where the two ovary walls fuse together. Similarly, at 3 weeks after anthesis, the fruit pericarp of NIL 34-18R is thicker because of increased cell size and number in the medio-lateral axis of the pericarp. Furthermore, fruit elongation in NIL 34-2 L is associated with a significant increase in cell elongation along the proximal-distal axis. Therefore, multiple processes, including cell expansion, cell division, and cell elongation, during the period of ovary and fruit growth, are affected by *fs10*.

The involvement of OFPs in regulating cell division and elongation has been demonstrated in multiple species. In tomato *ovate*, fruit elongation at the proximal end was associated with increased cell number along the proximal-distal axis (Wu et al., 2018). Whereas in tomato, the activity of *SlOFP20* was dependent on a mutation in *OVATE*, we did not observe such an interaction in pepper. This indicates that while the main effect of OFP members is mostly conserved in pepper and tomato, the interaction among OFPs may be species-specific. In rice (*Oryza sativa*), *GS9* acts as a transcriptional activator and regulator of grain elongation by altering cell division (Zhao et al., 2018). GS9 interacts with two OFPs, OsOFP14, and OsOFP8, and the latter interacts with OsGSK2 as a negative regulator of the brassinosteroid signaling pathway. Knockdown of *OsOFP6* by RNAi resulted in altered grain shape and reduced plant growth associated with a reduction in cell number, but with no effect on cell elongation (Ma et al., 2017). In *Arabidopsis*, a gain-of-function mutation in *AtOFP1* resulted in reduced length of the shoot and flower organs, and was associated with reduced cell elongation (Wang et al., 2007).

The *fs10* transcriptome-enrichment analysis revealed a large number of genes putatively associated with cell expansion; however, no enrichment was associated with cell division, despite the change in cell number in the ovaries of the QTL-NILs. This may be the result of gene activation related to cell division occurring at earlier stages of ovary development, i.e., before anthesis. Multiple hormone metabolism processes that are putatively associated with organ growth were enriched in the *fs10* transcriptome; however, there is no direct evidence linking the activity of specific differentially expressed genes with fruit shape determination. The major limitation of the transcriptome analysis in the present study was that RNA was sampled from a single narrow developmental stage, which precludes a more comprehensive assessment of gene-expression patterns during ovary and fruit development. Therefore, to study gene networks associated with *CaOFP20*, transcriptome analyses of multiple ovary and fruit developmental stages will be necessary.

## Data Availability Statement

The datasets presented in this study can be found in online repositories. The names of the repository/repositories and accession number(s) can be found at: NCBI BioProject - PRJNA783486.

## Author Contributions

YB, AR, AD-F, and HZ designed and conducted the experiments and analyzed the data. EK produced the plants. IP wrote the manuscript. All authors contributed to the article and approved the submitted version.

## Funding

Support for this research was provided by the G2P-SOL project and funded by the European Union Horizon 2020 research and innovation program under grant agreement no. 677379.

## Conflict of Interest

The authors declare that the research was conducted in the absence of any commercial or financial relationships that could be construed as a potential conflict of interest.

## Publisher’s Note

All claims expressed in this article are solely those of the authors and do not necessarily represent those of their affiliated organizations, or those of the publisher, the editors and the reviewers. Any product that may be evaluated in this article, or claim that may be made by its manufacturer, is not guaranteed or endorsed by the publisher.

## Footnotes

^1^ https://vanderknaaplab.uga.edu/tomato_analyzer.html

^2^ https://www.kyazma.nl/index.php/MapQTL/

^3^ http://www.g2p-sol.eu

^4^ https://bitbucket.org/tasseladmin/tassel-5-source/wiki/Tassel5GBSv2Pipeline

^5^ https://plantgarden.jp/en/list/t4072/genome/t4072.G002

^6^ http://peppergenome.snu.ac.kr/download.php

^7^ http://solgenomics.net

^8^ http://kobas.cbi.pku.edu.cn/kobas3/?t=1

^9^ https://broadinstitute.github.io/picard

^10^ http://www.hnivr.org/pepperhub
