# Supplementary material for: Pepper Fruit Elongation Is Controlled by Capsicum annuum Ovate Family Protein 20
Source: Front Plant Sci. 2022 Jan 4;12:815589. doi: 10.3389/fpls.2021.815589 (PMC8763684; doi:10.3389/fpls.2021.815589)
Supplement: Supplementary file 1 [file Data_Sheet_1.zip › Supplementary Material/Supplementary Table 1.DOCX]

**Supplementary Table S1.** Primers and markers used in the present study.

| SNP sequence  Ref/Alt | Primer sequence 5'→3' | Restriction  enzyme | SNP  position (Zunla-1) | Gene | Use | Primer name |
| --- | --- | --- | --- | --- | --- | --- |
| AGAAT/CTCCCA | GCTTGAGGAGGGGGAACAAC | EcoRI | 14856377 | Capana10g000455 | Marker | 14 Mbp_F |
|  | ATGATCCCTAGCTTCTGAAATCC |  |  |  |  | 14 Mbp_R |
| GACTG/TTTAAA | GTAGCCACAAACACCCTTGA | DraI | 20180904 | Capana10g000527 | Marker | 20 Mbp_F |
|  | TTGCGACAACCTCATTTACCTG |  |  |  |  | 20 Mbp_R |
| AGATT/CCTGCT | CAAGAGGAGATTGTTAGATATTTCAG | HinfI | 33139179 | Inter-genomic region | Marker | 33 Mbp_F |
|  | CTGACAAGCACTTCGACTCCTG |  |  |  |  | 33 Mbp_R |
| GATCT/CAAAGA | CCTGCTGAACTGGTTTCTTTAC | BamHI | 50323106 | Inter-genomic region | Marker | 50 Mbp_F |
|  | TACAGGAAAGGAAAGCAACCC |  |  |  |  | 50 Mbp_R |
| Indel of ~ 100 bp | GGGAGGAGCTTGTATACATCAA |  | 59550074 | Inter-genomic region | Marker | 60 Mbp_F |
|  | CAGTAGCACACAACGTCATCTCA |  |  |  |  | 60 Mbp_R |
| ATGTC/TCCAGG | GGGTATTAGGTCTTGCGAACAGT | BsaJI | 127817251 | Capana10g001216 | Marker | 127 Mbp_F |
|  | CAGGAGACGAGATGCCTTAC |  |  |  |  | 127 Mbp_R |
| TATTA/GAATCT | GAGACACACAAGCAACTCTTTT | MseI | 130159975 | Capana10g001230 | Marker | 130 Mbp_F |
|  | GAACGTCCACCATGCTCTTTAG |  |  |  |  | 130 Mbp_R |
| TTTAC/GGAGCA | ACATCCAACTGCTACGCCCT | MslI | 132429276 | Capana10g001247 | Marker | 132 Mbp_F |
|  | TAAAGAGGCGGCGAGCAGTA |  |  |  |  | 132 Mbp_R |
| ACCTT/GGGACT | CCCAACCCCACCATATCGCTCA | StyI | 139414835 | Capana10g001297 | Marker | 139 Mbp_F |
|  | GCACAACGTCGATTGAGGTGAAC |  |  |  |  | 139 Mbp_R |
| AGACC/AACAAT | CCCGGATAGTATCGCACTGAATTC | BsaI | 144799333 | Capana10g001354 | Marker | 144 Mbp_F |
|  | GCTTCTTGTTGGTTGGCGTCTTTGC |  |  |  |  | 144 Mbp_R |
| AGAAA/TCTATA | GAAATTGCGGTTGATTTTCCTGA | MwoI | 149238162 | Capana10g001384 | Marker | 149 Mbp_F |
|  | CCTGCATTTCACCATCACAAAAAG |  |  |  |  | 149 Mbp_R |
| GTGGC/AGCCAT | GACTCTGGGAAGGTTATGGAGC | HhaI | 156082302 | Capana10g001439 | Marker | 156 Mbp_F |
|  | CCTATCCTTATATAGTCACCGATTTC |  |  |  |  | 156 Mbp_R |
| CAAAC/TGCTAT | GTTGTATTTGCTTCCTGTTAATTATG | MslI | 159722252 | Capana10g001481 | Marker | 159 Mbp_F |
|  | CCAGACATCCTCTAAGATCAATCT |  |  |  |  | 159 Mbp_R |
| TTTCA/CCGGGT | GACCTTCTTTGTCCAGCATCG | XmaI | 165023066 | Capana10g001552 | Marker | 165 Mbp_F |
|  | CCTTCGCTGTTAAATTCTGATCC |  |  |  |  | 165 Mbp_R |
| CTCCG/AAAGAA | GCATCCTAATCCATCACCACATTCG | Hpy188I | 167918039 | Capana10g001614 | Marker | 167 Mbp_F |
|  | GGGAAACAGCCTCTCTACCTCCACG |  |  |  |  | 167 Mbp_R |
|  | CCATTTCACTGAGCCACCCA |  |  | Capana10g001230 | qRT-PCR | RT OFP20- F |
|  | GATAGGGACAAGGACGAGGAC |  |  |  |  | RT OFP20- R |
|  | GCACAAGCACAAGAAGGTTAAG |  |  | DQ975458 | qRT-PCR | UBQ RT-F |
|  | GCACCACACTCAGCATTAGGA |  |  |  |  | UBQ RT-R |
|  | GGAGAAAAGAGACACACAAGCAA |  |  | *CaOFP20* | Capana10g001230 ORF sequencing | OFP20_F |
|  | CGTCCACCATGCTCTTTAGT |  |  |  |  | OFP20_R |
|  | ATGGTACCTCGTCCTTGTCCCTATCCT |  |  |  | Cloning 296-bp fragment of Capana10g001230 into pTRV2 | OFP-Kpn-F |
|  | ATTCTAGAACTCTTCCTGCTTGCTTGA |  |  |  |  | OFP-Xba-R |
|  | TGGACTTAGATTCTGTGAGTAAGG  CCTAAAACTTCAGACACGGATCTACTT |  |  |  | pTRV2-specific primers | PYL156-F |
|  |  |  |  |  |  | PYL156-R |
|  | TGTGAGACTGCTACAATTAGGCG |  |  | *CaOVATE* | qRT-PCR of Capana02g002672 (*CaOVATE*) | Ca-Ovate RT-F |
|  | AGCTGGTAACTCGCTGTCTGA |  |  |  |  | Ca-Ovate RT-R |
| GCACG/ATCTTC | GCGTGAAAAGGAGAAATTAAACCG | HpyCH4IV | 148877881 | *CaOVATE* | Marker | Ovate-F |
|  | CACAAGAGGGAGCATATTGGAACG |  |  |  |  | Ovate-R |
| TTGTA/CGTGG | GGAGAAAAGAGACACACAAGCAA | Taq1 | 130160428 | *CaOFP20* | Marker for aa125 substitution | OFP20-F |
|  | TTTAGTCCAAACAGATTCAATAGACGCGCGACCTC |  |  |  |  | OFP dcaps373-TaqI-R |
| TATTA/GAATC | GGAGAAAAGAGACACACAAGCAA | MseI | 130160443 | *CaOFP20* | Marker for aa131substitution | OFP20-F |
|  | ACTAAAGAGCATGGTGGACG |  |  |  |  | OFP20-R |
| ACCCA/GAAAA | GTCCCTATCCTCTGACAAAATCCAGGCACCG | HpaII | 130160567 | *CaOFP20* | Marker for aa172  substitution | OFP dcapsHpaII-F |
|  | CATCAAGAAGGACAAGCGTTACAG |  |  |  |  | OFP dcaps-R |
| CGAC/CAAAA | GTCCCTATCCTCTGACAAAATCCAGGCACTC | Taq1 | 130160695 | *CaOFP20* | Marker for aa215  substitution | OFP dcapsTaqI-F |
|  | CATCAAGAAGGACAAGCGTTACAG |  |  |  |  | OFP dcaps-R |
| AGAGC/GATTG | GTCCCTATCCTCTGACAAAATCCAGGCACCG | MnlI | 130160728 | *CaOFP20* | Marker for aa225  substitution | OFPdcapsHpaII-F |
|  | ACTAAAGAGCATGGTGGACG |  |  |  |  | OFP20-R |
|  | GCTTCTCAAGGTGTAAAGTCG |  | 130121671 | *CaOFP20* | Primary PCR of the marker for OFP20 upstream InDel | OFP-1901del-F |
|  | GATAGTAGGGACAACTAAGATGACATG |  |  |  |  | OFP-1901del-R |
| InDel GAGTTATATTAAAGTAAAGAAGGAGGGTTTTATGATACACCA | GCTTCTCAAGGTGTAAAGTCG |  | 130121671 | *CaOFP20* | Secondary PCR of the marker for OFP20 upstream InDel | OFP-1901del-F1 |
|  | GATAGTAGGGACAACTAAGATGACATG |  |  |  |  | OFP-1901del-R1 |
