# Supplementary material for: Pepper Fruit Elongation Is Controlled by Capsicum annuum Ovate Family Protein 20
Source: Front Plant Sci. 2022 Jan 4;12:815589. doi: 10.3389/fpls.2021.815589 (PMC8763684; doi:10.3389/fpls.2021.815589)
Supplement: Supplementary file 1 [file Data_Sheet_1.zip › Supplementary Material/Supplementary Table 4.DOCX]

**Supplementary Table S4**. Expression pattern of *CaOFP20*. Data are derived for line 6421 from Pepperhub (<http://www.hnivr.org/pepperhub/>). Developmental stages used for measuring expression in the present study are marked in bold.

| Code | Capana10g001230 | Tissue |
| --- | --- | --- |
| L1 | 2.07±0.08 | Leaf |
| L2 | 1.87±0.68 | Leaf |
| L3 | 0.88±0.66 | Leaf |
| L4 | 1.25±0.49 | Leaf |
| L5 | 2.10±0.42 | Leaf |
| L6 | 4.45±0.09 | Leaf |
| L7 | 4.43±0.93 | Leaf |
| L8 | 2.50±0.98 | Leaf |
| L9 | 13.48±3.62 | Leaf |
| AL | 23.57±4.26 | Leaf/ABA |
| AR | 24.09±8.37 | Root/ABA |
| AS | 10.88±3.38 |  |
| F1 | 28.61±5.39 | Flower |
| F2 | 48.96±6.58 | Flower |
| F3 | 53.14±7.70 | Flower |
| F4 | 33.42±6.89 | Flower |
| F5 | 83.82±5.16 | Flower |
| F6 | 72.73±9.43 | Flower |
| F7 | 81.39±6.89 | Flower |
| F8 | 76.71±33.37 | Flower |
| F9 | 98.69±27.98 | Flower |
| P10 | 150.49±36.74 | Petal |
| O10 | 245.05±27.63 | **Ovary anthesis** |
| STA10 | 6.30±2.30 | Anther |
| FST0 | 76.02±5.71 | Whole fruit 3 DAP |
| FST1 | 78.46±13.64 | Whole fruit 7 DAP |
| G1 | 28.10±10.28 | Pericarp 10 DAP |
| G2 | 25.92±8.68 | Pericarp 15 DAP |
| G3 | 52.99±3.41 | **Pericarp 20 DAP** |
| G4 | 90.31±24.02 | Pericarp 25 DAP |
| G5 | 150.51±15.08 | pericarp 30 DAP |
| G6 | 141.55±38.37 | Pericarp 35 DAP |
| G7 | 10.60±4.73 | Pericarp 40 DAP |
| G8 | 2.92±1.20 | Pericarp 45 DAP |
| G9 | 2.42±0.49 | Pericarp 50 DAP |
| G10 | 1.81±1.15 | Pericarp 55 DAP |
| G11 | 3.24±3.34 | Pericarp 60 DAP |
| ST1 | 88.88±0.09 | Placenta and seeds 10 DAP |
| ST2 | 75.82±23.31 | Placenta and seeds 20 DAP |
| S3 | 54.54±21.35 | Seeds |
| S4 | 24.97±3.43 | Seeds |
| S5 | 12.12±1.93 | Seeds |
| S6 | 17.63±1.04 | Seeds |
| S7 | 27.24±3.67 | Seeds |
| S8 | 37.58±5.11 | Seeds |
| S9 | 33.90±8.95 | Seeds |
| S10 | 20.57±6.98 | Seeds |
| S11 | 18.18±7.95 | Seeds |
| T3 | 250.94±65.36 | **Placenta 20 DAP** |
| T4 | 685.35±68.99 | Placenta 25 DAP |
| T5 | 295.69±38.90 | Placenta 30 DAP |
| T6 | 176.78±23.68 | Placenta 35 DAP |
| T7 | 41.42±18.91 | Placenta 40 DAP |
| T8 | 8.08±3.08 | Placenta 45 DAP |
| T9 | 8.29±3.30 | Placenta 50 DAP |
| T10 | 4.82±1.10 | Placenta 55 DAP |
| T11 | 3.31±0.90 | Placenta 60 DAP |

ABA – abscisic acid; DAP – days after pollination.
