# Supplementary material for: Pepper Fruit Elongation Is Controlled by Capsicum annuum Ovate Family Protein 20
Source: Front Plant Sci. 2022 Jan 4;12:815589. doi: 10.3389/fpls.2021.815589 (PMC8763684; doi:10.3389/fpls.2021.815589)
Supplement: Supplementary file 1 [file Data_Sheet_1.zip › Supplementary Material/Supplementary Tables 2, 3, 7, 8.DOCX]

**Supplementary Figure S1** Cross sections of ovaries and fruit of *fs10*-NILs. **(A,B)** Ovaries at anthesis. **(C)** Fruit pericarp 3 weeks after anthesis. Red lines delineate the zones in which cellular measurements were performed in ovary walls and pericarps. Black lines across ovary walls in the proximal-distal direction indicate the axis for measurements of cell number and distance across the entire ovary wall length.

**Supplementary Figure S2** Enriched GO biological processes of differentially expressed genes between round (FSR)- and elongated (FSL)-fruited bulks (FDR ≤ 0.05). Left y axis: number of genes per enriched category (thick bars) and right y axis: significance level of each enriched category (thin line) in each bulk.

**Supplementary Table S1.** Primers and markers used in the present study.

**Supplementary Table S2.** Distribution of homozygous SNPs differing between round and elongated bulks identified by BSAseq in pepper chromosomes. The Zunla genome (Qin et al., 2014) was used for SNP mapping. Chromosome 00 – SNPs that were not assigned to chromosomes.

|  |  |  |  | Number of SNPs | Chromosome |
| --- | --- | --- | --- | --- | --- |
|  |  |  |  | 179 | 00 |
|  |  |  |  | 186 | 1 |
|  |  |  |  | 206 | 2 |
|  |  |  |  | 256 | 3 |
|  |  |  |  | 139 | 4 |
|  |  |  |  | 110 | 5 |
|  |  |  |  | 121 | 6 |
|  |  |  |  | 129 | 7 |
|  |  |  |  | 127 | 8 |
|  |  |  |  | 131 | 9 |
|  |  |  |  | 674 | 10 |
|  |  |  |  | 166 | 11 |
|  |  |  |  | 93 | 12 |
|  |  |  |  | 2517 | Total |
|  |  |  |  |  |  |

**Supplementary Table S3**. Shape indices of flower organs and leaves of *fs10-*NILs.

|  | Ovary shape index | Style shape index | Anther shape index | Petal shape index | Leaf shape index |
| --- | --- | --- | --- | --- | --- |
| NIL 34-2L | 0.98 ± 0.06 | 6.11 ± 1.17 | 2.36 ± 0.23 | 2.01 ± 1.07 | 2.04 ± 0.09 |
| NIL 34-18R | 0.73 ± 0.07 | 8.21 ± 1.67 | 1.77 ± 0.23 | 1.61 ± 0.16 | 2.23 ± 0.26 |
| *P* (*t*-test) | <0.0001 | 0.018 | 0.0006 | 0.003 | NS |

N = 7 flowers and leaves. NS – not significant. Means ± SD.

**Supplementary Table S4.** Expression pattern of *CaOFP20*. Data are derived for line 6421 from Pepperhub (<http://www.hnivr.org/pepperhub/>). Developmental stages used for measuring expression in the present study are marked in bold.

**Supplementary Table S5**. SNPs found in 50kbp upstream of the *CaOFP20* start codon and their association with Cis-acting regulatory DNA elements motifs.

**Supplemental Table S6**. Putative cis-acting regulatory DNA elements motifs located within the 42bp InDel.

**Supplementary Table S7**. Histological parameters of ovaries at anthesis of *fs10-*NILs (mean ± SD).

|  |  | NIL 34-2L | NIL 34-18R | *P*-value | N |
| --- | --- | --- | --- | --- | --- |
| Entire ovary | Cell number across longitudinal section | 176 ± 12.7 | 167 ± 13 | NS | 5 |
|  | Distance across longitudinal section (µm) | 3667.5 ± 362.6 | 3457.6 ± 562.6 | NS | 5 |
|  | Ovary length (µm) | 3201.3 ± 278.9 | 2920.64 ± 367 | NS | 5 |
|  | Ovary width (µm) | 2678.7 ± 243.2 | 3427.05 ± 175 | 0.0005 | 5 |
|  | Ovary shape index | 1.2 ± 0.11 | 0.8 ± 0.08 | 0.0008 | 5 |
| Longitudinal section  of ovary walls in segments 1,2,3* | Cell area (µm^2^) | 315.9 ± 35.1 | 481.3 ± 22.4 | <.0001 | 60* |
|  | Cell length (µm) | 27.91 ± 3.8 | 38.29 ± 2.8 | <.0001 | 300 |
|  | Cell width (µm) | 20.23 ± 3.4 | 26.29 ± 1.5 | <.0001 | 300 |
|  | Cell shape index | 1.38 ± 0.07 | 1.45 ± 0.09 | 0.03 | 300 |
|  | Cell layers across ovary wall section | 26.33 ± 2.5 | 28.46 ± 1.9 | 0.01 | 15 |
|  | Distance across ovary wall section (µm) | 586.03 ± 65.7 | 918.82 ± 111.2 | <.0001 | 15 |
|  | Cell area (µm^2^) | 389.80 ± 25.5 | 478.06 ± 20.8 | 0.0003 | 20* |
| Longitudinal section  of ovary wall in segment 4 | Cell length (µm) | 33.59 ± 3.2 | 43 ± 1.8 | 0.0005 | 100 |
|  | Cell width (µm) | 24.24 ± 1.4 | 27.61 ± 1.5 | 0.008 | 100 |
|  | Cell shape index | 1.38 ± 0.07 | 1.55 ± 0.04 | 0.002 | 100 |
|  | Cell layers across ovary wall section | 76.6 ± 10.1 | 98.7 ± 9.1 | <.0001 | 15 |
|  | Distance across ovary wall section (µm) | 1572.6 ± 258.6 | 2486.9 ± 278.6 | <.0001 | 15 |
|  | Cell area (µm^2^) | 361.67 ± 29.1 | 509.74 ± 22.9 | <.0001 | 20* |
| Transverse section  of ovary wall in segment 2 | Cell length (µm) | 31.64 ± 2.6 | 40.52 ± 3.6 | 0.002 | 100 |
|  | Cell width (µm) | 23.2 ± 2.4 | 28.67 ± 1.6 | 0.003 | 100 |
|  | Cell shape index | 1.37 ± 0.13 | 1.41 ± 0.1 | NS | 100 |
|  | Cell layers across ovary wall section | 24.9 ± 1.0 | 28.0 ± 0.5 | 0.001 | 15 |
|  | Distance across ovary wall section (µm) | 554.2 ± 45.3 | 847.5 ± 102.8 | <.0001 | 15 |

*Number of 100 μm x 100 μm squares. NS – not significant.

**Supplementary Table S8**. Histological parameters of fruit 3 weeks after anthesis of *fs10-*NILs (mean ± SD).

|  |  | NIL 34-2L | NIL 34-18R | *P*-value | N |
| --- | --- | --- | --- | --- | --- |
|  | Cell area (µm^2^) | 8960.7 ± 1097.7 | 8139.8 ± 1167.5 | NS | 60* |
| Longitudinal section  of pericarp | Cell length (µm) | 245.59 ± 25.5 | 202.12 ± 17.6 | <.0001 | 300 |
|  | Cell width (µm) | 97.87 ± 13 | 104.34 ± 14.9 | NS | 300 |
|  | Cell shape index | 2.53 ± 0.3 | 1.96 ± 0.2 | <.0001 | 300 |
|  | Cell layers across pericarp section | 30 ± 0.7 | 31.75 ± 2.8 | NS | 15 |
|  | Distance across pericarp section (µm) | 2150.8 ± 111.1 | 2489.69 ± 209.3 | 0.0025 | 15 |
|  | Cell area (µm^2^) | 6138.3 ± 881.6 | 7420.79 ± 1312.5 | 0.006 | 60* |
| Transverse section  of pericarp | Cell length (µm) | 153.23 ± 17.6 | 165.86 ± 20.2 | NS | 300 |
|  | Cell width (µm) | 113.34 ± 14.7 | 114.2 ± 13.2 | NS | 300 |
|  | Cell shape index | 1.35 ± 0.09 | 1.45 ± 0.1 | 0.02 | 300 |
|  | Cell layers across pericarp section | 28.85 ± 1 | 33 ± 1.8 | <.0001 | 15 |
|  | Distance across pericarp section (µm) | 2090.08 ± 238.4 | 2700.07 ± 263.1 | 0.0004 | 15 |

*Number of 500 μm x 500 μm squares. NS – not significant.

**Supplementary Table S9**. List of differentially expressed genes in the round and elongated bulks.
