# Supplementary figures and images for: Pepper Fruit Elongation Is Controlled by Capsicum annuum Ovate Family Protein 20
Source: Front Plant Sci. 2022 Jan 4;12:815589. doi: 10.3389/fpls.2021.815589 (PMC8763684; doi:10.3389/fpls.2021.815589)

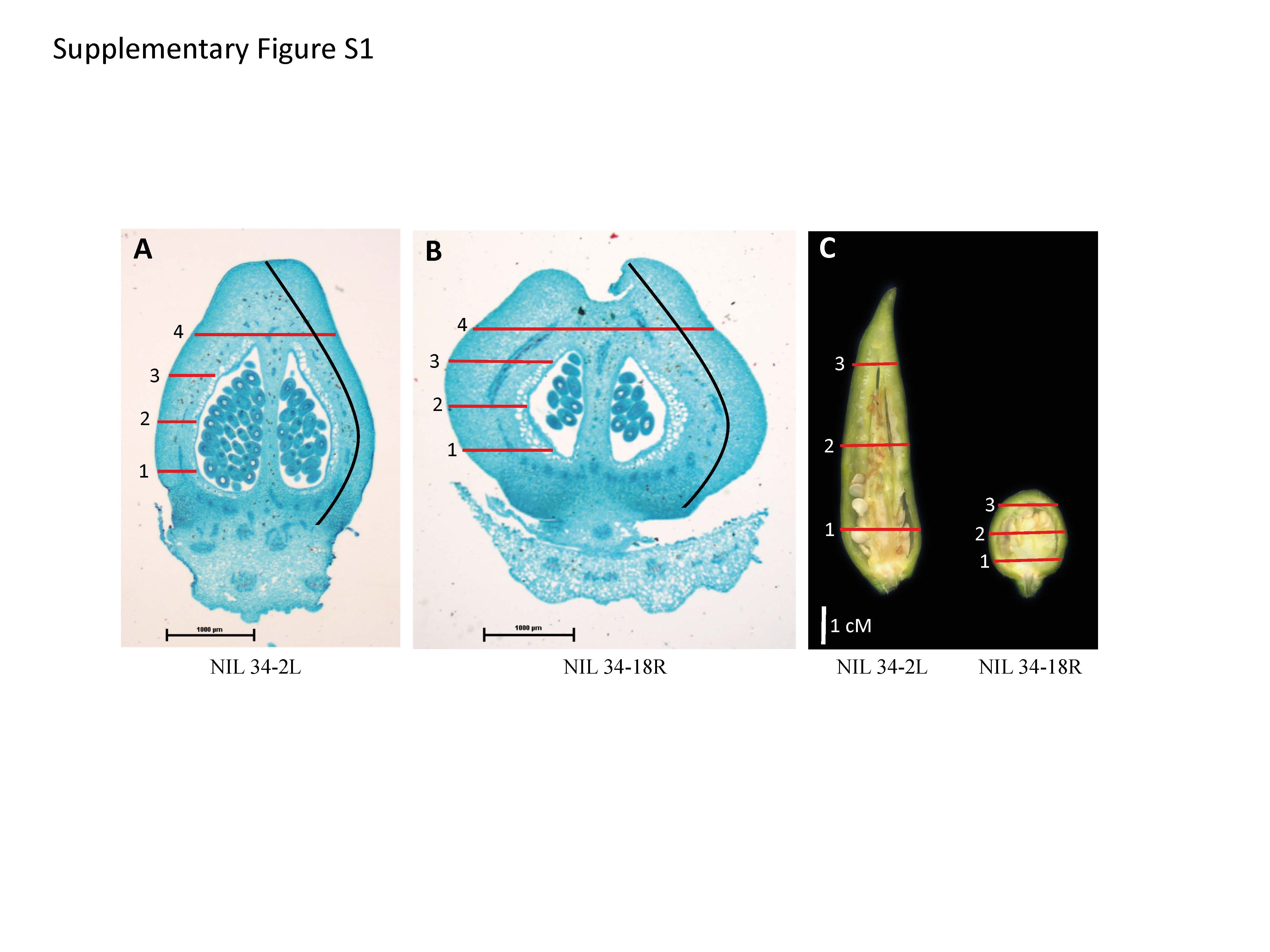

Supplement: Supplementary file 1 [file Data_Sheet_1.zip › Supplementary Material/Supplementary Figure 1.JPEG]

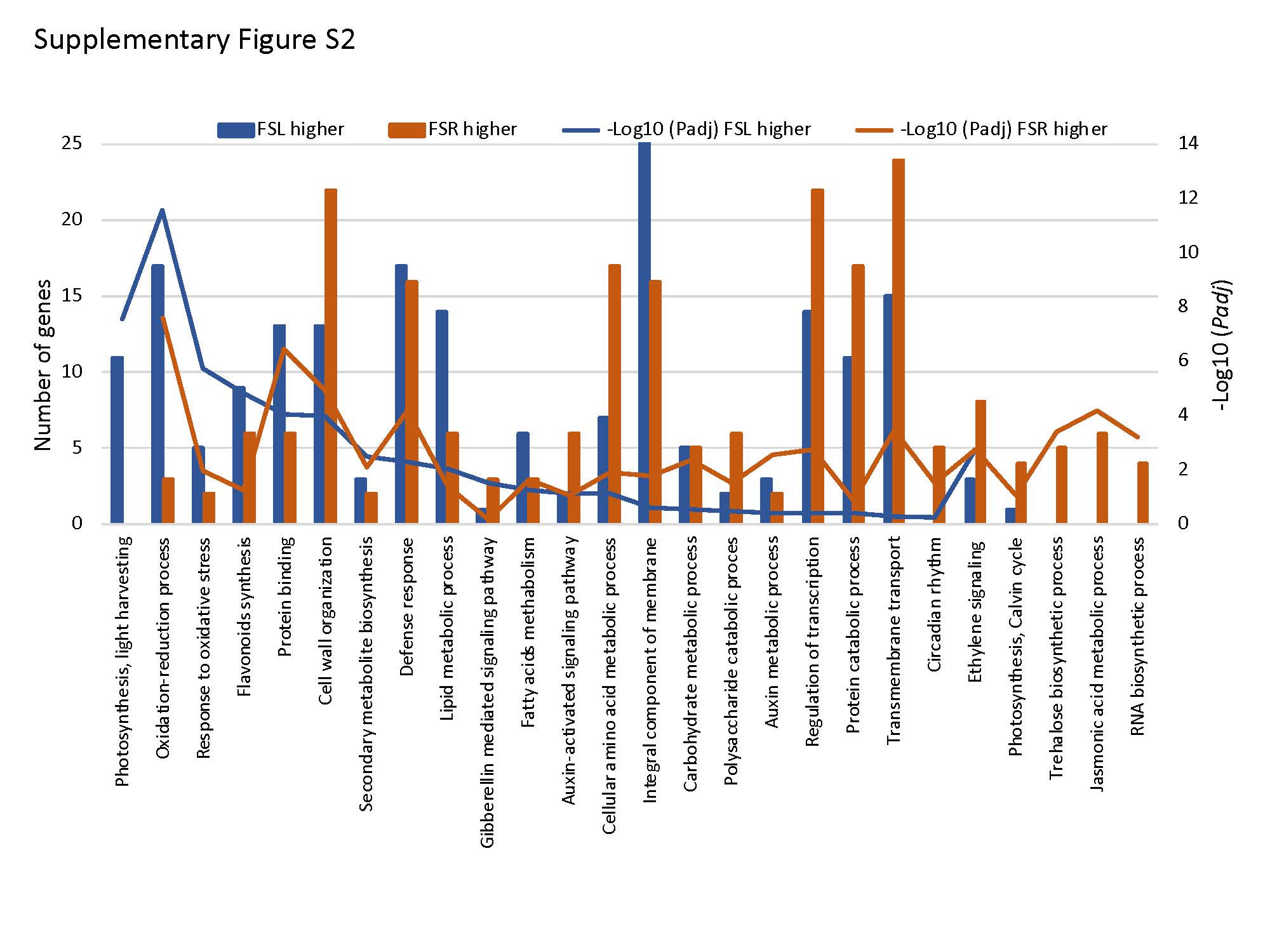

Supplement: Supplementary file 1 [file Data_Sheet_1.zip › Supplementary Material/Supplementary Figure 2.JPEG]
